# Risk for morbidity and mortality after neurosurgery in older patients with high grade gliomas – a retrospective population based study

**DOI:** 10.1186/s12877-022-03478-6

**Published:** 2022-10-17

**Authors:** David Löfgren, Antonios Valachis, Magnus Olivecrona

**Affiliations:** 1 Department of Oncology, Faculty of Medicine & Health, Örebro University, SE 70182 Örebro, Sweden; 2 Department of Neurosurgery, Faculty of Medicine & Health, Örebro University, SE 70182 Örebro, Sweden

**Keywords:** High grade glioma, Surgical complications, Elderly, Neurosurgical complications, Postoperative complications

## Abstract

**Background:**

Although high grade gliomas largely affect older patients, current evidence on neurosurgical complications is mostly based on studies including younger study populations. We aimed to investigate the risk for postoperative complications after neurosurgery in a population-based cohort of older patients with high grade gliomas, and explore changes over time.

**Methods:**

In this retrospective study we have used data from the Swedish Brain Tumour Registry and included patients in Sweden age 65 years or older, with surgery 1999–2017 for high grade gliomas. We analysed number of surgical procedures per year and which factors contribute to postoperative morbidity and mortality.

**Results:**

The study included 1998 surgical interventions from an area representing 60% of the Swedish population. Over time, there was an increase in surgical interventions in relation to the age specific population (*p* < 0.001).

Postoperative morbidity for 2006–2017 was 24%. Resection and not having a multifocal tumour were associated with higher risk for postoperative morbidity. Postoperative mortality for the same period was 5%. Increased age, biopsy, and poor performance status was associated with higher risk for postoperative mortality.

**Conclusions:**

This study shows an increase in surgical interventions over time, probably representing a more active treatment approach.

The relatively low postoperative morbidity- and mortality-rates suggests that surgery in older patients with suspected high grade gliomas can be a feasible option. However, caution is advised in patients with poor performance status where the possible surgical intervention would be a biopsy only.

Further, this study underlines the need for more standardised methods of reporting neurosurgical complications.

**Supplementary Information:**

The online version contains supplementary material available at 10.1186/s12877-022-03478-6.

## Background

The most common malignant primary brain tumours are the gliomas [1]. The most common gliomas are high grade gliomas (HGG) and specifically glioblastomas [1]. The median age of diagnosis in patients with glioblastomas or HGG is 59–65, with a higher incidence among older patients [1–4].

In adults, the estimated median survival with a glioblastoma is around 15 months but varies with age [2–4]. As an example, randomized studies regarding treatment on older populations have shown median survivals up to 9.7 months [5, 6].

Surgery is one of the therapy mainstays for HGG [7–9]. The aim of surgery is either to get a diagnostic biopsy or to make a resection as part of the treatment strategy. Neurosurgical procedures carry with them the risk of complications, among others new neurological deficits and risk for perioperative death. As a result, the decision on whether surgical treatment should be a part of the treatment strategy for each individual patient needs to consider these potential risks.

Although gliomas largely affect older patients, the current evidence on surgical procedures for gliomas and the potential risk for neurosurgical complications is mostly based on studies including younger study populations [2, 3, 10–14]. It is, therefore, essential to investigate the risk for complications after neurosurgical procedures in older patients with gliomas using real world data.

We aimed to investigate the risk for postoperative morbidity and mortality after neurosurgery in a nationwide population based cohort of older patients with HGG and to explore the potential changes in risks over time.

## Methods

### Study design

We performed a retrospective population- and registry-based study including all patients age 65 years or older, who had surgery for HGG between 1999 and 2017 in Sweden, using data from the Swedish Brain Tumour Registry (SBTR).

### The Swedish brain tumour registry

SBTR started in 1999, with the aim of collecting data on all patients that have undergone surgery for a primary brain tumour, on a nationwide level. Information has been collected about preoperative patient characteristics, tumour data and the postoperative course. The registry has, historically, had an almost complete coverage in three of the six Swedish healthcare regions [3]. During later years, one more healthcare region has retrospectively registered data from 1999 to 2017 to an almost complete coverage and is thus being accepted into this study as the fourth high coverage region out of the six healthcare regions in Sweden [15]. This corresponds to an approximate coverage of 60% of the Swedish population covering both rural and city areas [16].

### Study cohort

We have included all patients with HGG in the SBTR, from the four high coverage regions. HGG were selected using the SNOMED classification data reported to the registry and was defined as glioblastoma (9440/X, 9441/X), gliosarcoma (9442/X), astrocytoma gr III (9401/X), malignant glioma (9380/X), and gliomatosis cerebri (9381/X). SNOMED coding table is available as Supplementary information (Table 1). Not included as HGG were Oligodendroglioma grade III in conjunction with suggestions from the Swedish National Brain Tumour Trialist Group, and Oligoastrocytomas since the SNOMED code is identical for Oligoastrocytomas grades II and III.

To be included in the study cohort, surgery had to have been performed between 1999 and 2017. We chose 65 years of age as cut-off for being considered old, which is as a commonly used definition, and thus used as inclusion criteria in this study [5, 6, 17, 18]. We included all surgical interventions registered (both primary and secondary) as separate events.

All data regarding the demography of the healthcare regions are from official sources at Statistiska centralbyrån (Statistics Sweden) [16].

### Variables

Variables extracted from the SBTR, years of surgery they are available in the registry, and variable characteristics are available as Supplementary information (Table 2). Due to major changes in the SBTR regarding use of variables 1999–2005 versus 2006–2017, we chose 2005/2006 as a cut-off for baseline comparisons.

From the registry, patient sex and age at surgery were determined and used in creating 5-year age groups and for comparative analysis.

Date of death is included in SBTR directly from the Swedish Tax Agency.

For the variable Tumour size, information was available only for the period 2006–2015. Tumour size was defined as the largest diameter on radiological examination and was possible to input as:< 4 cm, 4-6 cm, or > 6 cm. For Tumour site, variables from 1999 to 2005 and 2006–2017 differs largely but the variable Multifocal tumour was available during the entire study period. Multifocal tumour was defined in the registry as radiologically distinct separate tumour components, even within a single lobe. We have used combined information from available variables to create the variable Tumour location (defined as multifocal or other).

The variable Preoperative symptoms is a combination of the three preoperative symptom variables (epilepsy, focal neurological symptoms and symptoms of intracranial pressure) and their parent variable No signs of symptoms. Since only the variable preoperative focal neurological symptoms was available 1999–2005, this has not been compared between the periods.

Type of surgery was specified in three categories 1999–2015 (biopsy, resection and radical resection) and with an added fourth category (near radical resection) from 2016. These were grouped as biopsy or resection (any type) in univariate and multivariate analysis.

WHO/ECOG performance status (WHO-PS) was recorded for the registry prior to surgery [19]. We have used the reported score for preoperative WHO-PS to form three groups: 0–1, 2, and 3–4.

### Outcome variables

The postoperative variables Local infection, Local hematoma and Thromboembolism were available 1999–2005 with the addition from 2006 of New seizures, New focal deficits and Reoperation. Because of these changes only the later study period was examined further. According to registry definitions Local infection represents a deep or superficial infection adjacent to the surgical area and Local hematoma represents an intracranial bleeding. The variable Postoperative morbidity (representing any complication) was created from the available postoperative complication variables and used as outcome variable in all morbidity calculations.

In the registry, according to registry instructions, all complications registered are within the first 30 days after date of surgery.

In this study all deaths within the first 30 days from surgery were defined as postoperative mortality.

### Statistics

We present age as medians and interquartile range. Variance in age between the periods was analysed using Mann-Whitney U test. Variables with categorical data were summarized using descriptive statistics and analysed with Pearson’s χ ^2^-test when possible.

Crude risk estimates with odds ratios (OR), confidence intervals (CI) and *p*-values for the outcomes were calculated using univariate logistic regression. The analysis of surgery per year used logistic regression with Performed surgery (yes/no) as the dependent variable and year of surgery as independent variable.

In multivariate analyses, we used a logistic regression model to calculate adjusted ORs and their corresponding CI for each outcome variable with the following pre-defined independent variables (entered simultaneously): age, sex, preoperative symptoms present, tumour size, type of surgery, tumour site (multifocal/other), WHO-PS groups and year of surgery.

Due to the different uses of variables in various periods, only data from years 2006–2015 were used for univariate and multivariate analyses.

IBM SPSS Statistics for Windows, Version 25.0, Armonk, NY, USA, was used for all statistical calculations. Microsoft Excel 2016 was used for initial sorting, calculating legal sex, date of birth and for calculating time from surgery to date of death. Statistical significance level was set to *p* < 0.05 and all CIs are at the 95% confidence level.

## Results

### Study cohort

**Fig. 1:**
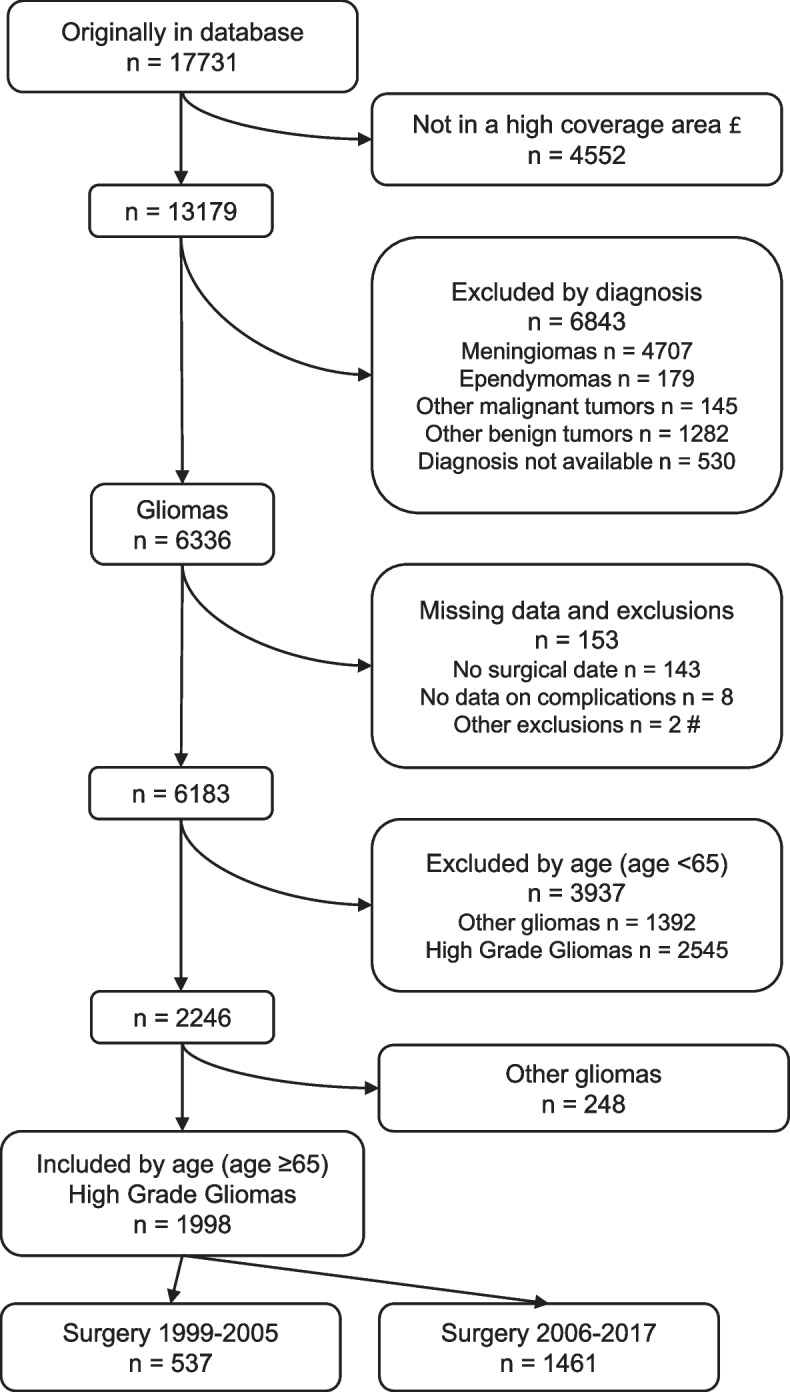
Study flowchart. Data selection and reasons for exclusion. £ - as described in Methods; # - date of surgery superseded by official date of death

We initially retrieved 17,731 records for primary intracranial CNS-tumours from the SBTR (available as Supplementary information, Table 1). The final study cohort included 1998 surgical interventions. Data selection and reasons for exclusion are depicted in Fig. 1. SNOMED distribution in the final study cohort is available as Supplementary Information (Table 3).

Characteristics of the study population are available in Table 1. The study population was significantly older during the later surgical period, both in terms of median age (*p* < 0.001) and distribution in different age groups (*p* = 0.004). Female/male ratio was approximately 1:1.5 (41%, *n* = 820 female and 59%, *n* = 1178 male) and remained stable throughout the entire study period (*p* = 0.234).

**Table 1:** Baseline characteristics

			Year of surgery	
**Variable**		**Total**	**1999–2005**	**2006–2017**
**Total number of patients**	n	1998	537	1461
**Age**	Median (IQR)	71 (68–75)	70 (67–74)	71 (68–75)
**Age groups**	n (% of surgical year)			
65–69		789 (39.5)	237 (44.1)	552 (37.8)
70–74		665 (33.3)	179 (33.3)	486 (33.3)
75–79		432 (21.6)	104 (19.4)	328 (22.5)
80+		112 (5.6)	17 (3.2)	95 (6.5)
**Sex**	Female	820 (41.0)	232 (43.2)	588 (40.2)
	Male	1178 (59.0)	305 (56.8)	873 (59.8)
**Tumour Size**	n (% of valid)			*n* = 1005 ^a^
< 4 cm			N/A	392 (39.0) ^a^
4–6 cm			N/A	452 (45.0) ^a^
> 6 cm			N/A	161 (16.0) ^a^
**Tumour Site**	n (% of valid)	*n* = 1898	*n* = 449	*n* = 1449
Other		1476 (77.8)	376 (83.7)	1100 (75.9)
Multifocal		422 (22.2)	73 (16.3)	349 (24.1)
**Preoperative symptoms**	n (% of valid per variable)			
Any symptoms present			N/A	1437 (98.4)
Focal deficit			387 (73.6)	1152 (79.5)
Seizures			N/A	375 (26.1)
Symptoms of intracranial pressure			N/A	613 (42.7)
**Type of surgical intervention**	n (% of valid)		*n* = 535	*n* = 1452
Type of surgery (biopsy only)			119 (22.2)	537 (37.0)
			*n* = 535	*n* = 1103 ^a^
*Biopsy*			119 (22.2)	405 (36.7) ^a^
*Resection*			271 (50.7)	352 (31.9) ^a^
*Radical resection*			145 (27.1)	346 (31.4) ^a^
**WHO/ECOG Performance status**	n (% of valid)	*n* = 1959	*n* = 522	*n* = 1437
	0	462 (23.6)	150 (28.7)	312 (21.7)
	1	625 (31.9)	161 (30.8)	464 (32.3)
	2	563 (28.7)	140 (26.8)	423 (29.4)
	3	243 (12.4)	45 (8.6)	198 (13.8)
	4	66 (3.4)	26 (5.0)	40 (2.8)
By group	0–1	1087 (55.5)	311 (59.6)	776 (54.0)
	2	563 (28.7)	140 (26.8)	423 (29.4)
	3–4	309 (15.8)	71 (13.6)	238 (16.6)
**Postoperative morbidity**	n (% of valid per variable)			
Any complication			62 (11.5)	355 (24.3)
Local infection			12 (2.2)	64 (4.4)
Local hematoma			43 (8.0)	118 (8.1)
Thromboembolism			16 (3.0)	58 (4.0)
New seizures			N/A	63 (4.3)
New focal deficit			N/A	179 (12.3)
Reoperation			N/A	62 (4.3)
*Cause for reoperation*	n (% of Reoperations)			*n* = 62
*Reoperation and local infection*			N/A	15 (24.2)
*Reoperation and local hematoma*			N/A	20 (32.3)
*Reoperation, local infection and hematoma*			N/A	4 (6.5)
**Postoperative mortality**		120 (6.0)	49 (9.1)	71 (4.9)

*n* numbers, *IQR* Interquartile range, *N/A* Data not Available

^a^years 2006–2015

There were more cases with multifocal tumours undergoing surgery during the later surgical period (*p* < 0.001) but due to the changes in reporting no other comparisons regarding tumour site were made.

Nearly all patients (2006–2017) had one or more preoperative symptom/−s (*n* = 1437, 98.4%). From those with specified symptoms, 53.3% (*n* = 766) had one, 38.8% (*n* = 557) had two and 5.8% (*n* = 83) had three preoperative symptoms reported.

Type of surgical intervention could not be evaluated in detail due to the changes made in reporting. Biopsy only was more common during the later study period when compared to resection of any type (*p* < 0.001). Only 0.3% (*n* = 5) of patients had a second surgical event, 3 of these patients had both their surgical interventions at an age of 65 years and above. All of these events were during the later study period.

Preoperative WHO-PS was available for 98% of all patients with no statistically significant difference for the WHO-PS groups (*p* = 0.075) between the early and late surgical period.

**Fig. 2:**
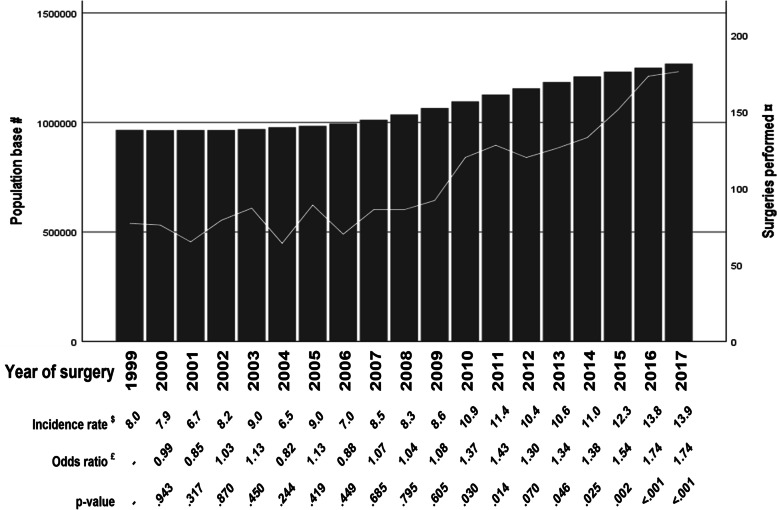
Population base and number of surgeries by year of surgery. Number of persons, age 65 y or older living in the studied healthcare regions and number of surgeries performed, incidence rate, OR for having surgery, and corresponding *p*-value by year of surgery. # - n of persons, age 65 y or older (bars); ¤ - n of surgeries performed (line); $ - incidence rate of surgery per 100,000 for each year; £ - OR for having surgery from univariate logistic regression with 1999 as index year. *p*-value for the regression < 0.001

Number of surgical interventions per year and the population base for each year is reported in Fig. 2. There was a statistically significant increase in surgeries performed on this age group over time (*p* < 0.001).

### Postoperative morbidity

The proportion of patients suffering from postoperative morbidity for the early and late study periods was 11.5% (*n* = 62) and 24.3% (*n* = 355) respectively. In the late study period 15.6% (*n* = 228) had one, 5.7% (*n* = 83) had two and 3.0% (*n* = 44) had three or more postoperative complications registered.

Distribution of postoperative complications in relation to type of surgical intervention is available as Supplementary Information (Table 4).

Table 2 shows a summary of OR for the outcome postoperative morbidity. In the unadjusted model the variables sex, type of surgery, tumour site and year of surgery showed a statistically significant association with the outcome.

**Table 2:** Postoperative morbidity

Variable	CRUDE OR (CI)	***p***-value	ADJUSTED OR (CI)	***p***-value
**Age at surgery**	0.976 (0.946–1.007)	0.122	0.992 (0.957–1.028)	0.645
**Sex (f/m)**	1.433 (1.062–1.934)	0.018	1.374 (0.976–1.934)	0.068
**Type of surgery**				
Biopsy vs other	2.395 (1.715–3.345)	< 0.001	2.130 (1.446–3.137)	< 0.001
		< 0.001^#^		
*Biopsy* vs *resection*	2.420 (1.666–3.514)	< 0.001		
*Biopsy* vs *radical resection*	2.370 (1.629–3.449)	< 0.001		
**WHO Performance status**		0.503^#^		0.283^#^
0–1 vs 2	1.202 (0.871–1.659)	0.264	1.336 (0.934–1.913)	0.113
0–1 vs 3–4	1.158 (0.772–1.737)	0.479	1.170 (0.714–1.917)	0.533
**Preoperative symptoms (No vs Yes)**				
Symptoms present	2.508 (0.578–10.886)	0.220	1.645 (0.359–7.539)	0.522
*Focal deficit*	1.190 (0.820–1.726)	0.360		
*Seizures*	0.828 (0.590–1.163)	0.276		
*Symptoms of ICP*	1.236 (0.918–1.664)	0.163		
**Tumour site**				
Multifocal tumour vs other	1.665 (1.142–2.426)	0.008	1.586 (1.003–2.508)	0.048
**Tumour size**		0.081^#^		0.101^#^
< 4 cm vs 4-6 cm	0.877 (0.623–1.235)	0.453	0.854 (0.594–1.227)	0.392
< 4 cm vs > 6 cm	1.421 (0.927–2.178)	0.107	1.390 (0.877–2.203)	0.162
**Year of surgery**		0.003^#^		0.054^#^
2006 vs 2007	1.459 (0.542–3.931)	0.455	1.012 (0.356–2.877)	0.983
2006 vs 2008	2.382 (0.933–6.086)	0.070	1.641 (0.613–4.394)	0.324
2006 vs 2009	2.662 (1.061–6.681)	0.037	2.112 (0.819–5.447)	0.122
2006 vs 2010	1.909 (0.767–4.752)	0.165	1.635 (0.640–4.179)	0.304
2006 vs 2011	2.294 (0.940–5.596)	0.068	2.210 (0.886–5.517)	0.089
2006 vs 2012	3.135 (1.298–7.568)	0.011	2.617 (1.054–6.494)	0.038
2006 vs 2013	1.598 (0.636–4.013)	0.318	1.277 (0.494–3.301)	0.614
2006 vs 2014	3.600 (1.513–8.565)	0.004	2.809 (1.152–6.846)	0.023
2006 vs 2015	4.067 (1.732–9.549)	0.001	2.767 (1.018–7.523)	0.046

Odds ratio and 95% confidence interval for postoperative morbidity

*ICP* Intracranial pressure

^*#*^variable *p*-value

In the adjusted model, only type of surgery (other than biopsy) and tumour site (other than multifocal) made an independent statistically significant contribution to the outcome. Hosmer and Lemeshow test for goodness of fit renders *p* = 0.103 indicating support for this model.

### Postoperative mortality

In the early study period (1999–2005) 9.1% (*n* = 49) died within 30 days of surgery. During the later period (2006–2017) postoperative mortality was statistically significantly lower at 4.9% (*n* = 71), *p* < 0.001. Only 35.2% (*n* = 25) of deaths within the postoperative period during 2006–2017 were recorded having a postoperative complication. The registered complication with highest proportion of mortality was reoperation due to side effects with 12.2% (*n* = 6) followed by postoperative localized hematoma with 12.0% (*n* = 9), new or worsened focal neurological deficit or symptoms with 10.4% (*n* = 14), new or worsened seizures with 8.7% (*n* = 4), local postoperative infection with 4.9% (*n* = 2) and thromboembolism with 3.7% (*n* = 1).

As evident in Table 3, both the unadjusted and the adjusted model showed increased age, biopsy as type of surgery, and high WHO-PS (WHO-PS 3–4) as having statistically significant association to the outcome. Hosmer and Lemeshow test for goodness of fit shows support for the adjusted model (*p* = 0.991).

**Table 3:** Postoperative mortality

Variable	CRUDE OR (CI)	***p***-value	ADJUSTED OR (CI)	***p***-value
**Age at surgery**	1.078 (1.022–1.136)	0.006	1.070 (1.005–1.139)	0.035
**Sex (f/m)**	1.180 (0.688–2.024)	0.547	1.455 (0.773–2.737)	0.245
**Type of surgery**				
Other vs Biopsy	3.715 (2.140–6.450)	< 0.001	2.575 (1.341–4.944)	0.004
		< 0.001^#^		
*Resection* vs *Biopsy*	2.646 (1.414–4.950)	0.002		
*Radical resection* vs *Biopsy*	6.211 (2.597–14.925)	< 0.001		
**WHO Performance status**		0.001^#^		0.003^#^
0–1 vs 2	2.497 (1.260–4.949)	0.009	2.023 (0.964–4.249)	0.063
0–1 vs 3–4	5.518 (2.774–10.974)	< 0.001	4.034 (1.827–8.908)	0.001
**Preoperative symptoms (Yes vs No)**				
Symptoms present	1.981 (0.449–8.742)	0.367	3.714 (0.744–18.544)	0.110
*Focal deficit*	0.724 (0.351–1.497)	0.384		
*Seizures*	3.086 (1.310–7.265)	0.010		
*Symptoms of ICP*	0.852 (0.494–1.470)	0.565		
**Tumour site**				
Other vs Multifocal tumour	1.758 (0.994–3.107)	0.052	1.324 (0.661–2.652)	0.428
**Tumour size**		0.777^#^		0.385^#^
< 4 cm vs 4-6 cm	1.135 (0.623–2.067)	0.678	1.135 (0.586–2.199)	0.707
< 4 cm vs > 6 cm	0.845 (0.350–2.040)	0.709	0.575 (0.209–1.584)	0.284
**Year of surgery**		0.132^#^		0.363^#^
2006 vs 2007	0.470 (0.108–2.039)	0.313	0.534 (0.084–3.417)	0.508
2006 vs 2008	1.907 (0.630–5.774)	0.254	2.087 (0.503–8.654)	0.311
2006 vs 2009	0.907 (0.265–3.102)	0.876	1.443 (0.332–6.273)	0.625
2006 vs 2010	0.929 (0.292–2.957)	0.900	0.922 (0.218–3.893)	0.912
2006 vs 2011	0.752 (0.230–2.464)	0.638	0.994 (0.237–4.167)	0.993
2006 vs 2012	0.333 (0.077–1.440)	0.141	0.483 (0.091–2.570)	0.394
2006 vs 2013	0.317 (0.073–1.369)	0.124	0.319 (0.059–1.736)	0.186
2006 vs 2014	0.614 (0.181–2.088)	0.435	0.908 (0.211–3.911)	0.897
2006 vs 2015	0.727 (0.229–2.309)	0.589	0.952 (0.193–4.708)	0.952

Odds ratio and 95% confidence interval for postoperative mortality

*ICP* Intracranial pressure

^#^variable *p*-value

## Discussion

This retrospective, registry and population based study shows a distinct increase in surgery performed on patients 65 years or older with HGG over time. This observation might reflect a more active approach to the treatment of older patients with HGG in later years, and is not only due to an increase in the age specific population. Interestingly, this trend does not come, according to our data, at the price of more postoperative mortality nor morbidity. Year of surgery does not have a significant influence on neither of these outcomes in the adjusted regression models. The increase in multifocal tumours and the increase of biopsy only as surgical procedure during the later study period might indicate that this group of patients are being selected for surgery (diagnostic biopsy) to a higher degree in the later years of the study. This hypothesis is strengthen by the very low incidence rate of surgery during most of the studied years in comparison with reported age specific incidence rates for the largest HGG subgroup, the glioblastomas [20–22]. This study does not have information on patients with a possible glioma that has not undergone surgery and it is known from modern material with an overlapping age group that there may be as much as 35% having radiological features of glioblastoma without histological verification [23]. Because of this, we would discourage from using our results as an indication of increased incidence of high grade gliomas.

### Postoperative morbidity

We have shown an overall risk of postoperative morbidity within 30 days of surgery of 24% for the period 2006–2017 using 6 different subtypes of complications. In comparison, reported results include complication rates ranging from 6% [24] to 68% [25] for mixed ages and using different definitions of complications. As examples from older HGG patient materials Karsy et al. demonstrated 32% overall complication rates including patients 75 years or older whereas Almenawer et al. indicated 6.6–13.3% morbidity in their meta-analysis with patients 60 years or older [26, 27]. This wide range of reported complications implicates the need for a standardized method of classification and reporting of postoperative complications and adverse outcomes.

In our study we analysed what factors contribute to the risk of postoperative complications, knowing that these sometimes lead to shorter survival and less postoperative treatment [10, 12, 26]. Only having a resection of any kind and having a tumour that was not multifocal increased the risk according to our adjusted logistic regression model. Since this is not a randomized study, these results might be explained by selection bias or by a most likely, co-variation between resection and non-multifocal tumours. Having a resection instead of a biopsy has been identified as a risk factor for complications in other materials [28]. We could not detect any association between increase in postoperative morbidity and preoperative WHO-PS in contrast to findings in other studies suggesting poor performance status or frailty as a risk factor for this outcome [28–30]. Our findings are, however, in line with those of Karsy et al. with a similar (older) age group showing no difference in median preoperative Karnofsky-score between patients with and without complications [26]. Cloney et al. showed that frailty but not Karnofsky-score was associated with poor outcome [29]. This suggests the need for a more comprehensive frailty assessment than performance status in order to predict postoperative complications in older age groups.

### Postoperative mortality

In this material, we have a postoperative 30-day mortality of 6% in total with a statistically significant decrease from 9 to 5% when comparing 1999–2005 with 2006–2017.

There is great variation in postoperative mortality reported in different studies [31]. Our result with an overall postoperative mortality of 5% for the years 2006–2017 in an older population is somewhat higher but within the range of those reported in other studies with younger populations [24, 30–37]. As with many of these studies, we have reported overall death within 30 days and not death from verified surgical or surgery-related complications. Using patients dead within 30 days with registered complications, as surgery related mortality, gives us numbers similar to those of De Witt Hamer et al. who reported 37% of early deaths as being related to surgery and Graus et al. who reported 58% of postoperative deaths as related to postoperative complications [31, 36].

High WHO-PS, having a biopsy instead of other types of resection, and higher age at surgery were associated with a higher risk of postoperative mortality.

Our results indicate that patients with WHO-PS 3–4 were 4 times more likely than patients with WHO-PS 0–1, to die within the postoperative period. The importance of performance status or frailty for the risk of postoperative mortality are in line with findings in other studies [29, 30, 32, 38]. Surprisingly, nearly 16% of patients in our material had a WHO-PS of 3–4 (corresponding to a Karnofsky grade of 40 or worse) [19]. These patients had a considerably high rate of postoperative mortality, in excess of 14%, a fact that needs to be considered in the decision-making process.

Our findings regarding type of surgery are in line with the findings of Graus et al. and Almenawer et al. demonstrating biopsy as associated with higher postoperative mortality but conflicting with the findings of De Witt Hamer et al. showing that hospital percentage of biopsies was not significantly associated with early mortality [27, 31, 36].

Age is a known risk factor for postoperative mortality in other previous studies and our results could claim to confirm this [13, 32, 34, 37].

### Strengths and limitations

There are several strengths and limitations with this study that should be considered when translating the results into daily clinical practice.

The population based database including all or nearly all patients in a larger geographical area covering multiple neurosurgical clinics is one of the study’s strengths. As described by Skaga et al., only a selected minority of glioblastoma or HGG-patients are usually represented in clinical trials, adding to the value of real world data from population based studies [14]. Although only 974 patients were included in the multivariate analysis part of the study, it remains one of the larger studies covering postoperative morbidity and mortality in older patients with HGG [24, 27, 30–37].

A major limitation of this study is the fact that the variables in use for postoperative complications lacks information about grading and duration of the complications. We can hypothesise that a registered complication has to have had some level of impact on the patient but the dichotomous grading of complications might be too coarse compared to available grading systems like that proposed by Dindo and Clavien making comparisons of our findings regarding postoperative morbidity with other studies more difficult [39, 40].

Another potential limitation is the lack of a more comprehensive method of determining frailty other than using WHO-PS as a surrogate, since frailty may be associated with poorer outcomes independent of performance status [29]. Furthermore, the SBTR lacks information regarding other co-morbidities, concomitant medications and other essential factors needed for commonly used validated frailty or comorbidity scoring system [17, 41–43]. WHO-PS (or other performance-status grading systems) is, however, commonly used as exclusion criterion in randomized clinical oncology trials and WHO-PS > 2 is a common cut-off for treatment-recommendations in oncology, justifying it’s use in this study [5, 6, 19].

The SBTR has a high grade of coverage verified against the official national cancer registry and spans over nearly two decades [3, 15]. Even though there is a good coverage of pre- and postoperative variables recorded in the SBTR, an important aspect influencing our analyses is the fact that not all variables were available for all years due to changes in report forms, thus limiting the available cases for thorough examination. This is an obvious problem for many clinical registries that span over many years and cover a range of different treatment strategies over time. The treatment for older patients with HGG was in many aspects different in 1999 than the treatment strategies in 2017 [6, 44]. Nevertheless, for this study, we have been able to take advantage of the official population data and the long period of coverage in the registry, despite the use of different variables, in the calculations regarding number of surgical interventions in relation to the population and in the multivariate analysis for postoperative morbidity and mortality.

## Conclusions

This study shows an increase in surgical interventions over time on patients 65 years or older with HGG, probably representing a more active treatment approach by the Swedish neuro-oncology society.

Using the results in this study, we can conclude that surgery in the older patient with suspected HGG is possible and can be a feasible option. We suggest caution, especially with preoperative WHO-PS of 3–4 where the planned or possible surgical intervention would be a biopsy only.

This study further underlines the need, and use for a more standardised method of reporting and classifying complications from neurosurgery.

## Supplementary Information

**Additional file 1.**

## Data Availability

The datasets generated during and/or analysed during the current study are not publicly available due to their sensitive personal nature but are available from the corresponding author on reasonable request.

## Abbreviations

CI: Confidence intervals
ECOG: Eastern Cooperative Oncology Group
HGG: High grade glioma
OR: Odds ratios
SBTR: Swedish Brain Tumour Registry
WHO: World Health Organization
WHO-PS: WHO/ECOG Performance status
