# Supplementary material for: Risk for morbidity and mortality after neurosurgery in older patients with high grade gliomas – a retrospective population based study
Source: BMC Geriatr. 2022 Oct 17;22:805. doi: 10.1186/s12877-022-03478-6 (PMC9575213; doi:10.1186/s12877-022-03478-6)
Supplement: Supplementary file 1 — Additional file 1. [file 12877_2022_3478_MOESM1_ESM.docx]

# Supplementary information

Risk for morbidity and mortality after neurosurgery in older patients with high grade gliomas – a retrospective population based study.

Table 1

| SNOMED coding table, all available codes in the Swedish Brain Tumour Registry (SBTR) | | | |
| --- | --- | --- | --- |
|  |  |  |  |
| **M-code** | **Morphology term, commonly used** | **Diagnosis group in study** | **N in SBTR** |
| 33400 | Cyst | Other benign tumours | 21 |
| 71620 | Colloid cyst | Other benign tumours | 8 |
| 72900 | Cholesteatoma | Other benign tumours | 5 |
| 80000 | Tumour, benign | Other benign tumours | 3 |
| 80001 | Tumour, unknown malignancy | Other benign tumours | 13 |
| 80003 | Tumour, malignant NOS | Other malignant tumours | 6 |
| 80203 | Carcinoma, undifferentiated, NOS | Other malignant tumours | 1 |
| 80213 | Carcinoma, anaplastic, NOS | Other malignant tumours | 1 |
| 81403 | Adenocarcinoma, NOS | Other malignant tumours | 2 |
| 82463 | Neuroendocrine carcinoma, NOS | Other malignant tumours | 2 |
| 88500 | Lipoma, NOS | Other benign tumours | 1 |
| 90643 | Germinoma | Other malignant tumours | 21 |
| 90800 | Teratoma, benign | Other benign tumours | 3 |
| 90803 | Teratoma, malignant | Other malignant tumours | 2 |
| 90840 | Dermoid cyst, NOS | Other benign tumours | 25 |
| 91200 | Hemangioma | Other benign tumours | 56 |
| 91501 | Hemangiopericytoma, unknown malignancy | Other benign tumours | 8 |
| 91503 | Hemangiopericytoma, malignant | Other malignant tumours | 11 |
| 91611 | Hemangioblastoma | Other benign tumours | 215 |
| 93501 | Craniopharyngioma | Other benign tumours | 102 |
| 93601 | Pinealoma | Other benign tumours | 11 |
| 93623 | Pineoblastoma | Other malignant tumours | 9 |
| 93630 | Melanotic neuroectodermal tumor | Other malignant tumours | 2 |
| 93801 | Glioma of uncertain malignant potential | Glioma, High grade group | 4 |
| 93803 | Glioma, malignant | Glioma, High grade group | 361 |
| 93813 | Gliomatosis cerebri | Glioma, High grade group | 5 |
| 93823 | Oligoastrocytoma gr II/III | Glioma, other | 315 |
| 93831 | Subependymoma | Ependymomas | 59 |
| 93841 | Subependymal giant cell astrocytoma | Glioma, other | 11 |
| 93900 | Choroid plexus papilloma, NOS | Other benign tumours | 25 |
| 93901 | Atypical choroid plexus papilloma | Other malignant tumours | 3 |
| 93903 | Choroid plexus carcinoma | Other malignant tumours | 1 |
| 93913 | Ependymoma, NOS | Ependymomas | 132 |
| 93923 | Ependymoma, anaplastic | Ependymomas | 26 |
| 93933 | Papillary ependymoma | Ependymomas | 1 |
| 93941 | Myxopapillary ependymoma | Ependymomas | 1 |
| 94001 | Diffuse astrocytoma, of uncertain malignant potential | Glioma, other | 2 |
| 94003 | Diffuse astrocytoma, low grade | Glioma, other | 690 |
| 94013 | Astrocytoma, anaplastic | Glioma, High grade group | 709 |
| 94103 | Protoplasmic astrocytoma | Glioma, other | 1 |
| 94113 | Gemistocytic astrocytoma | Glioma, other | 56 |
| 94121 | Desmoplastic infantile astrocytoma | Glioma, other | 1 |
| 94130 | Dysembryoplastic neuroepithelial tumor | Other benign tumours | 21 |
| 94203 | Fibrillary astrocytoma | Glioma, other | 60 |
| 94211 | Pilocytic astrocytoma | Glioma, other | 107 |
| 94213 | Pilocytic astrocytoma (94211 in ICD-O-3) | Glioma, other | 60 |
| 94223 | Spongioblastoma, NOS (94211 in ICD-O-3) | Glioma, other | 1 |
| 94243 | Pleomorphic xanthoastrocytoma | Glioma, other | 16 |
| 94253 | Pilomyxoid astrocytoma | Glioma, other | 2 |
| 94401 | Does not exist in ICD-O-2/3, treated as 94403 | Glioma, High grade group | 1 |
| 94403 | Glioblastoma, NOS | Glioma, High grade group | 5055 |
| 94413 | Giant cell glioblastoma | Glioma, High grade group | 78 |
| 94423 | Gliosarcoma | Glioma, High grade group | 122 |
| 94503 | Oligodendroglioma, NOS | Glioma, other | 456 |
| 94513 | Oligodendroglioma, anaplastic | Glioma, other | 308 |
| 94603 | Oligodendroblastoma | Glioma, other | 5 |
| 94703 | Medulloblastoma, NOS | Other malignant tumours | 45 |
| 94713 | Desmoplastic nodular medulloblastoma | Other malignant tumours | 7 |
| 94733 | Primitive neuroectodermal tumor, NOS | Other malignant tumours | 67 |
| 94743 | Large cell medulloblastoma | Other malignant tumours | 3 |
| 94900 | Ganglioneuroma | Other benign tumours | 4 |
| 94903 | Ganglioneuroblastoma | Other malignant tumours | 1 |
| 94920 | Gangliocytoma | Other benign tumours | 5 |
| 94930 | Dysplastic gangliocytoma of cerebellum | Other benign tumours | 3 |
| 95003 | Neuroblastoma, NOS | Other malignant tumours | 7 |
| 95013 | Medulloepithelioma, NOS | Other malignant tumours | 1 |
| 95030 | Does not exist in ICD-O-2/3, treated as 94130 | Other benign tumours | 8 |
| 95033 | Neuroepithelioma, NOS | Other malignant tumours | 3 |
| 95050 | Ganglioglioma, benign | Glioma, other | 1 |
| 95051 | Ganglioglioma, NOS | Glioma, other | 112 |
| 95053 | Ganglioglioma, anaplastic | Glioma, other | 8 |
| 95060 | Central neurocytoma, benign | Other benign tumours | 3 |
| 95061 | Central neurocytoma | Other benign tumours | 26 |
| 95083 | Atypical teratoid/rhabdoid tumor | Other malignant tumours | 1 |
| 95223 | Olfactory neuroblastoma | Other malignant tumours | 26 |
| 95233 | Olfactory neuroepithelioma | Other malignant tumours | 1 |
| 95300 | Meningioma, NOS | Meningiomas | 4543 |
| 95301 | Meningiomatosis, NOS | Meningiomas | 30 |
| 95303 | Meningioma, malignant | Meningiomas | 73 |
| 95310 | Meningothelial meningioma | Meningiomas | 526 |
| 95320 | Fibrous meningioma | Meningiomas | 207 |
| 95330 | Psammomatous meningioma | Meningiomas | 63 |
| 95340 | Angiomatous meningioma | Meningiomas | 74 |
| 95370 | Transitional meningioma | Meningiomas | 276 |
| 95381 | Clear cell meningioma | Meningiomas | 41 |
| 95383 | Papillary meningioma | Meningiomas | 8 |
| 95391 | Atypical meningioma | Meningiomas | 450 |
| 95393 | Meningeal sarcomatosis | Other malignant tumours | 3 |
| 95400 | Neurofibroma, NOS | Other benign tumours | 5 |
| 95403 | Malignant peripheral nerve sheath tumor | Other malignant tumours | 3 |
| 95500 | Plexiform neurofibroma | Other benign tumours | 1 |
| 95600 | Neurilemoma, NOS | Other benign tumours | 1177 |
| 95603 | Neurilemoma, malignant | Other malignant tumours | 1 |
|  |  |  |  |
|  |  | Sum - SNOMED code | 17036 |
| 99 |  | Sum - no info | 618 |
| Alter |  | Sum - "Alternative missing" | 77 |
|  |  |  |  |
|  |  | Sum - SNOMED +missing | 17731 |

Table 2

| **Study Variables** |  |  |
| --- | --- | --- |
| **Variable name** | **Years available** | **Definition/explanation** |
| Personal Identity Number | 1999-2017 | Containing date of birth and sex |
| Date of surgery | 1999-2017 |  |
| Date of death | 1999-2017 | Automatically imported to registry |
| WHO Performance status | 1999-2017 | Preoperative performance status (0-4) |
| **Preoperative symptoms** |  |  |
| Focal neurological symptoms | 1999-2017 | Preoperative symptoms caused by tumour |
| No signs of symptoms | 2006-2017 | Parent variable indicating no preoperative symptoms |
| Epilepsy | 2006-2017 | Preoperative seizures caused by tumour |
| Symptoms of intracranial pressure | 2006-2017 | Preoperative symptoms caused by tumour |
| **Tumour site** |  |  |
| Multifocal tumour | 1999-2017 | Multifocal growth pattern |
| Bilateral | 2006-2017 | Bilateral tumour growth |
| Right side | 1999-2017 | Growth in right hemisphere |
| Left side | 1999-2017 | Growth in left hemisphere |
| Frontal lobe | 1999-2005 |  |
| Temporal lobe | 1999-2005 |  |
| Parietal lobe | 1999-2005 |  |
| Occipital lobe | 1999-2005 |  |
| Cerebellum | 1999-2005 |  |
| Cerebellopontine Angle | 1999-2005 |  |
| Posterior cranial fossa | 2006-2017 |  |
| Base of skull | 1999-2017 |  |
| Brain stem | 1999-2005 |  |
| Central location | 1999-2017 | Thalamus, basal ganglia etc. |
| **Tumour size** | 2006-2015 | Size of tumour by largest diameter (<4cm ; 4-6cm ; >6cm) |
| **Type of surgical intervention** |  |  |
| Biopsy | 1999-2017 | Diagnostic biopsy only |
| Resection | 1999-2017 | Extensive surgery, not radical |
| Radical resection | 1999-2017 | Extensive surgery, gross total resection |
| Near radical resection | 2016-2017 | Extensive surgery, small residual tumour |
| **Postoperative complications** |  |  |
| Local infection | 1999-2017 | Postoperative local infection |
| Local hematoma | 1999-2017 | Postoperative intracranial bleeding |
| Thromboembolism | 1999-2017 | Postoperative thromboembolism, any kind |
| Postoperative complications | 2009-2010 | Parent variable, any type of complication |
| New seizures | 2006-2017 | New or worsened seizures |
| New focal deficit | 2006-2017 | New or worsened focal neurological deficits |
| Reoperation | 2006-2017 | Reoperation due to complications |
| **SNOMED tumour code** | 1999-2017 | Systematized Nomenclature of Medicine, morphology |
| Study variables from the SBTR, with variable explanation and years available on report forms. | | |

Table 3

| SNOMED Distribution in the final study cohort | | | |
| --- | --- | --- | --- |
|  |  |  |  |
| **M-code** | **Morphology term, commonly used** | **N in final study cohort** | |
| 9380/X | Glioma, malignant |  | 104 |
| 9381/X | Gliomatosis cerebri |  | 2 |
| 9401/X | Astrocytoma, anaplastic |  | 166 |
| 9440/X | Glioblastoma |  | 1669 |
| 9441/X | Giant cell glioblastoma |  | 16 |
| 9442/X | Gliosarcoma |  | 41 |
|  |  |  |  |
|  |  | Sum | 1998 |

Table 4

| Postoperative morbidity by type of surgery | |  |  |
| --- | --- | --- | --- |
|  | **Type of surgery** | |  |
|  | **Biopsy** | **Resection** | **p-value** |
| Any complication | 84 (15.6) | 269 (29.4) | <0.001 |
| Local infection | 12 (2.2) | 51 (5.6) | 0.003 |
| Local hematoma | 23 (4.3) | 95 (10.4) | <0.001 |
| Thromboembolism | 8 (1.5) | 50 (5.5) | <0.001 |
| New seizures | 18 (3.4) | 45 (4.9) | 0.156 |
| New focal deficit | 41 (7.6) | 137 (15.0) | <0.001 |
| Reoperation | 14 (2.6) | 48 (5.3) | 0.016 |
| Number and percentage of valid with complication for each variable, years 2006-2017 | | | |
